# Nanomaterial Characterization in Complex Media—Guidance and Application

**DOI:** 10.3390/nano13050922

**Published:** 2023-03-02

**Authors:** Yves Uwe Hachenberger, Daniel Rosenkranz, Charlotte Kromer, Benjamin Christoph Krause, Nadine Dreiack, Fabian Lukas Kriegel, Ekaterina Koz’menko, Harald Jungnickel, Jutta Tentschert, Frank Stefan Bierkandt, Peter Laux, Ulrich Panne, Andreas Luch

**Affiliations:** 1 Department of Chemical & Product Safety, German Federal Institute for Risk Assessment (BfR), Max-Dohrn-Strasse 8-10, 10589 Berlin, Germany; 2 Institute for Clinical Chemistry and Laboratory Medicin, Klinikum Oldenburg AöR, Rahel-Straus-Straße 10, 26133 Oldenburg, Germany; 3 Federal Institute for Materials Research and Testing (BAM), Richard-Willstätter-Strasse 11, 12489 Berlin, Germany

**Keywords:** nano, characterization, spICP-MS, matrix, dissolution

## Abstract

A broad range of inorganic nanoparticles (NPs) and their dissolved ions possess a possible toxicological risk for human health and the environment. Reliable and robust measurements of dissolution effects may be influenced by the sample matrix, which challenges the analytical method of choice. In this study, CuO NPs were investigated in several dissolution experiments. Two analytical techniques (dynamic light scattering (DLS) and inductively-coupled plasma mass spectrometry (ICP-MS)) were used to characterize NPs (size distribution curves) time-dependently in different complex matrices (e.g., artificial lung lining fluids and cell culture media). The advantages and challenges of each analytical approach are evaluated and discussed. Additionally, a direct-injection single particle (DI sp)ICP-MS technique for assessing the size distribution curve of the dissolved particles was developed and evaluated. The DI technique provides a sensitive response even at low concentrations without any dilution of the complex sample matrix. These experiments were further enhanced with an automated data evaluation procedure to objectively distinguish between ionic and NP events. With this approach, a fast and reproducible determination of inorganic NPs and ionic backgrounds can be achieved. This study can serve as guidance when choosing the optimal analytical method for NP characterization and for the determination of the origin of an adverse effect in NP toxicity.

## 1. Introduction

Nanomaterials (NMs) exhibit a unique size-to-surface ratio, which leads to novel properties of the NMs [1,2,3]. Examples of some relevant inorganic NMs are metal nanoparticles (NPs) made of gold (Au) [4] or silver (Ag) [5] or metal oxide NPs [6] such as Al${}_{2}$O${}_{3}$ [7], CeO${}_{2}$ [8] or CuO [9]. Due to their advantages compared to bulk materials, NPs experience an increase in application in a broad range of products, such as food packaging [10], textiles [11], agriculture [12], cosmetics [13] and pharmaceuticals [14].

This development leads to safety concerns about this emerging group of functional materials [15,16,17]. The research in this area reveals that a broad range of parameters of the NPs have an influence on the outcome of NP-related toxicological studies. On the one hand, different intrinsic physico-chemical parameters of the NPs, such as size [18] and shape [19], as well as surface properties, such as charge [20] or chemical modifications [21], have an influence on the fate of the NPs and any successive biological response [22]. On the other hand, additional interactions of the NPs after their suspension, e.g., with specific media or artificial liquid constituents, may modify the NP behavior in the dispersion (e.g., agglomeration and sedimentation, dissolution or stabilization) [23]. A prominent example of this phenomenon is the formation of a protein corona around NPs in a cell culture medium [24]. It is crucial to identify potential risks of dissolving NPs in an exposure scenario to evaluate health risks, especially for metal oxides [25,26,27]. An important aspect is the differentiation between cellular responses generated from particulate effects and those generated by dissolved ions, which might be generated at prolonged exposure [28,29]. When these considerations are taken into account, determining the source of NP toxicity becomes increasingly difficult. Nevertheless, this challenge has to be solved in order to estimate and evaluate potential health risks in different NP application scenarios.

A frequently used technique to obtain NP size distribution curves is dynamic light scattering (DLS). In short, the scatter profile of the sample dispersion, based on the brownian motion, is measured over time and converted to a correlation function, which can be used to calculate size distribution curves. Reasons for the broad application of DLS measurements for NP characterization are their easy accessibility, high versatility, and fast procedures for the measurement of the hydrodynamic diameter of the particles [30]. However, the characterization of polydisperse NPs, as well as interference in complex media, are documented challenges of this technique [31].

Due to its sensitivity and selectivity, the inductively-coupled plasma mass spectrometry used in single particle mode (spICP-MS) is another established technique to characterize metal and metal oxide NPs [32]. Assuming a spherical shape, the size of the metal or metal oxide can be derived for a known particle composition of nearly all metals [33]. An additional analytical challenge to this point is the complex matrix, which is present in most human organs, such as the lung, as well as in toxicological studies. These matrix effects are a major concern for the analytical investigation of real-life samples. Additional sample preparation steps, such as dilutions or digestion steps, are necessary for these complex matrices [34,35,36]. Nevertheless, these steps often lead to other problems. For example, diluting low-concentrated dispersions may lead to number concentrations falling short of detection limits, and digestion steps will alter the sample directly, possibly affecting the NPs. In particular, the digestions may, in the worst case, lead to the loss of individual NP information. Another approach to overcome such challenges is the application of direct nebulization strategies with low volumes [37].

To evaluate the capabilities of a method, the robustness is important, especially in terms of different kinds of samples and their compounds. To establish a method that is able to tackle diverse complex media, this study used a wide array of increasingly complex aqueous media to mimic such scenarios. A selection of solutions and media applied in dissolution and toxicity experiments were used to take a broad range of matrix effects into account. An important point to mention is the risk of these complex systems interfering with the individual measurement techniques. Milli-Q water was used as a blank medium without additional effects. To avoid pH-driven dissolution, a sodium hydrocarbonate buffer was used [38]. As a first step toward complex cell culture medium, a solution containing a set Bovine Serum Albumin (BSA) concentration was used [39]. Here, observing bigger hydrodynamic sizes is to be expected due to the protein corona formation around the NPs [40]. This effect should be further enhanced in Roswell Park Memorial Institute (RPMI) cell culture media. In this media, the additional ionic components may influence the size of the formed corona as well as the fate and behavior of the NPs. On top of that, the buffered solution should counteract pH-driven dissolution activities. An artificial model system of the air–lung interface is “Gamble’s solution” (GS), an ionic model for the lung lining surfactant [41]. The influence of ionic components should be even stronger here. An enhanced version of Gamble’s solution (EGS) is a modified one with additionally dispersed lipids, which are closer to a real-life situation [41]. Lastly, artificial lysosomal fluid (ALF) is used to emulate an environment comparable to the one inside lysosomes, which are natural storage and digestion sites within single cells for nanoparticles. This medium simulates an intracellular scenario after a possible particle uptake [41]. In this case, the acidic pH, which is present in lysosomes, is expected to further destabilize the NPs.

In this study, metal oxide NPs made of CuO were used as a model system due to their broad size distribution and pH-driven dissolution properties [42]. In combination with the model matrices, these samples were used to evaluate the capabilities of different techniques (DLS, microwave digestion with ICP-MS and direct-injection (DI) spICP-MS) in challenging scenarios. Measurements were conducted every hour for the first 8 h and then after 1, 2 and 7 days. The high frequency of time points at the start of the experiment takes into account the potential higher reactivity and aims to reduce possible errors based on these. A DI spICP-MS method was developed and optimized to directly measure small volumes of the sample without the requirement of additional preparation steps. The development process was based on the technical specifications ISO TS 19590:2017 [43]. The approach developed in this study can be used as a fast method to determine inorganic NPs and their potential health risks attributed to the dissolution and subsequent ion formation in a broad range of complex matrices at low sample concentrations. Additionally, these findings contribute to the progress of biomodelling and dosimetry approaches to close the gap between in vivo and in vitro results. Thus, the results deepen the understanding of NP toxicity.

## 2. Materials and Methods

### 2.1. Chemicals and Materials

If not noted, otherwise stated chemicals were purchased at Merck (Darmstadt, Germany) with at least 98% purity. The sample handling was performed in 50 mL tubes (Corning 430828, Corning, New York, NY, USA). Stock solutions of copper oxide NPs (544868, Merck, Darmstadt, Germany) were prepared in accordance with the Nanogenotox protocol: “Final protocol for producing suitable manufactured NMs exposure media” (October 2011). Shortly, a 2.56 mg mL${}^{-1}$ stock dispersion was freshly prepared by pre-wetting the powder with 0.5% (*v*/*v*) ethanol (96%) followed by the addition of Milli-Q water (MilliPore gradient, Merck, Darmstadt, Germany) containing 0.05% BSA. The dispersion was sonicated for 5 min and 9 s at an amplitude of 10% with a probe sonifier (200 W Bandelin Sonopuls HD 2200, BANDELIN Electronic GmbH & Co. KG, Berlin, Germany). The sample was cooled in an ice-water bath during sonication [44].

For the enhanced Gamble’s solution, 1,2-dipalmitoyl-sn-glycero-3-phosphocholine (16:0 PC; DPPC) was provided by Avanti Polar Lipids, Inc. (850355C via Merck, Darmstadt, Germany). To ensure a fine dispersion of DPPC, a sonication was performed as described in the Nanongenotox protocol after its addition. The different media were prepared according to the literature [41]. As a commonly used cell culture medium, RPMI 1640 (Merck, Darmstadt, Germany) was used. Similar to a normal use case, 10% fetal bovine serum and 1% antibiotics (10,000 µg mL${}^{-1}$ streptomycin and 10,000 units mL${}^{-1}$ penicillin) were added to the RPMI. The freshly prepared NP stock solutions were further diluted with regard to the different techniques. Final concentrations of 25 mg L${}^{-1}$, 20 µg L${}^{-1}$ and 1 µg L${}^{-1}$ in the media were used for the DLS measurements, for the microwave digestions and for the direct measurements, respectively. Standard solutions of dissolved Au and Cu (1000 mg L${}^{-1}$, TraceCERT, Sigma-Aldrich, Darmstadt, Germany), 3.5% nitric acid (technical grade 70% *v*/*v*, VWR, Darmstadt, Germany) were purified in a douPur quartz sub-boiling point apparatus (MLS GmbH, Leutkirch im Allgäu, Germany), and the other media were used for the calibration solutions (1, 2, 5 and 10 µg L${}^{-1}$). The reference Au-NPs NIST 8012 (30 nm, Gaithersburg, MD, USA) were diluted in the respective media to concentrations down to 50 ng L${}^{-1}$ to determine matrix-matched transport efficiencies of the DI spICP-MS set-up.

### 2.2. Experimental Details

All samples were stored at 20 °C. At defined time points (approx. at 0, 1, 2, 4, 6, 8, 24, 48 and 168 h) samples of CuO NP dispersions inside the different media were characterized with DLS after vortexing the samples.

The sample preparation for the acid-assisted microwave digestions consisted of a vortexing step, after which a volume of 1 mL was extracted into an Eppendorf tube. Afterwards, the samples were centrifuged at 200 rpm for 5 min to force the sedimentation of the NPs. Directly following this step, the top 500 µL of the samples were separated, and the pellet inside the leftover solution was redispersed through vortexing. As a next step, the solutions were digested by adding 69% HNO${}_{3}$ and 30% H${}_{2}$O${}_{2}$ and processing them at 200 °C and 160 bars. Afterward, a 1:50 dilution with Milli-Q water was performed prior to the ICP-MS measurements.

In the case of DI spICP-MS, measurements were performed more frequently (between 15 min to 1 h) to a total of 20–30 time points per experiment. Each experiment was performed with three independent replicates and additional media blanks.

### 2.3. Instrumentation and Data Evaluation

The DLS analysis was performed with a Zetasizer from Malvern Panalytics (Kassel, Germany). Thermal equilibration time was set to 60 s at 25 °C. Each value and time point represents the average of five individual DLS measurements using automatic optimization of analytical conditions and data treatment by general-purpose size analysis. NP sizes were determined as z-average and intensity mean. Furthermore, the polydispersity index (PDI) and the count rate were visualized. Bar plots of these values average the measurements below 12 h against the determined averages after several days (i.e., 1–7). Therefore, the relative standard deviation (RSD) is influenced by the uncertainty of the individual measurements and the processes over time.

For the evaluation of ionic contents of the digested solutions, measurements were performed with a quadrupole ICP mass spectrometer (iCAP Q, Thermo Fisher Scientific GmbH, Dreieich, Germany) equipped with a PFA ST Nebulizer, a quartz cyclonic spray chamber and a 2.5 mm quartz injector (all from Thermo Fisher Scientific). The gas flow for the cool gas (Ar) and the auxiliary gas (Ar) were set to 14 and 0.65 L min${}^{-1}$, respectively. The sample flow rate was determined to be 0.34 mL min${}^{-1}$. ${}^{59}$Co and ${}^{65}$Cu were analyzed using the collision cell technique at 5 mL min${}^{-1}$ (collision gas flow with 93% He and 7% H${}_{2}$).

Measurements of the CuO NPs were performed with the ${}^{65}$Cu signal to avoid possible interferences from salts. Due to the complete dissolution of NPs as part of the microwave digestion, the ionic contents of the supernatant and the sedimented NPs were evaluated. These values were averaged to the total ionic content, and the particle-based part was calculated by taking into account the measured ionic concentration of the supernatant. In addition to the bar plots with long and short-term values, the ionic distributions at the individual time points were visualized to highlight the different influences of the media on the NP dissolution.

For single particle analysis of the NP solutions, a quadrupole ICP-MS (Thermo Scientific XSERIES II, Thermo Fisher Scientific, Waltham, MA, USA) with a PFA ST Nebulizer, a quartz cyclonic spray chamber and a 2.5 mm quartz injector (all from ESI Elemental Service and Instruments GmbH, Mainz, Germany) were used. Using the time-resolved analysis mode for data acquisition, intensities were collected as a function of time (counts per dwell-time interval). The acquisition time for each run was set to 70 s with a dwell time (or data acquisition rate) of 3 ms. The gas flows for the plasma, the nebulizer and the auxiliary (all Ar) were set to 13, 0.89 and 0.7 L min${}^{-1}$, respectively. All measurements were performed using the collision cell technique to avoid polyatomic interferences with the same collision gas and flow rates as mentioned before. The injection system was a modified microFAST MC system (ESI Elemental Service and Instruments GmbH, Mainz, Germany) with a single loop (300 µL) set-up and 2 independent syringes (5 mL and 500 µL). A sample loop of 300 µL was used with a flow rate of 500 µL min${}^{-1}$ to ensure a transient signal independent from the media composition. The measurement scripts were adjusted in accordance with the set-up characteristics and to reduce the overall measurement time. Routine tuning of this system was performed with an adapted routine and the commonly used Tune F solution (ESI Elemental Service and Instruments GmbH, Mainz, Germany) to maximize the signal intensity of ${}^{59}$Co. Results of an aqueous 5 µg L${}^{-1}$ Au solution, as described above, were used to evaluate the performance at different flow rates (50, 75, 100, 150, 300 and 500 µL min${}^{-1}$) based on the averaged duration, intensity and its RSD.

Further experiments were performed with a flow rate of 500 µL min${}^{-1}$ to avoid the clogging effects of higher viscosity media. For the evaluation of these parameters and the determination of matrix-matched transport efficiencies, the ${}^{197}$Au signal was observed. The determination of the threshold between ionic background and particle signals and the respective counting of the observed particle number were performed systematically, based on the corrected average intensities of the ionic background in an automated fashion. First, the transient signal of the individual data set was divided into 10 consecutive pieces (typically 1000 data points each) to take drifts during a single measurement into account. Next, the total average value and average of each piece was determined.

To determine the threshold, either 3 or 5 times the RSD (σ) was added to each average [45,46]. The individual point values above their respective limit were taken out and the whole procedure was repeated 5 times. The final limit of each timepiece was used to determine the particle number of each data piece, respectively. The averaged particle number per measurement was used to determine the matrix-matched transport efficiency.

Similar to the automated approach described above, the particle numbers, as well as the particle size and ionic background, were calculated at each time point. The basis of this approach is the excel spreadsheet from RIKILT Wageningen UR available at https://www.wur.nl/en/show/Single-Particle-Calculation-tool.htm (accessed on 21 September 2021). To obtain the total copper content for the visualization similar to the microwave digestions, the ionic content and particulate content were summed up. The particle numbers were corrected using the matrix-specific transport efficiencies, and the concentration was derived based on the actual volume used.

## 3. Results and Discussion

### 3.1. Nanomaterial Characterization of Hydrodynamic Parameters in Complex Media

**Figure 1:**
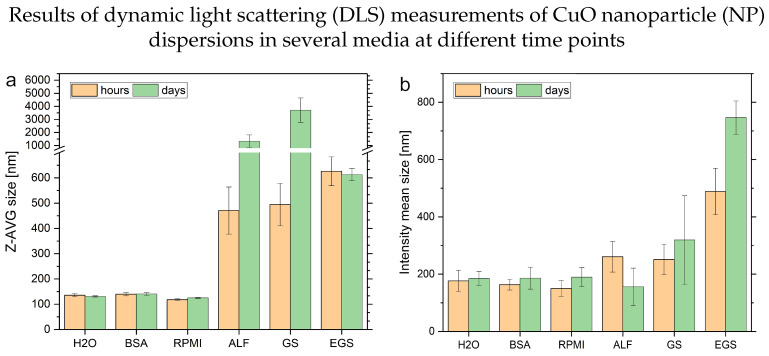

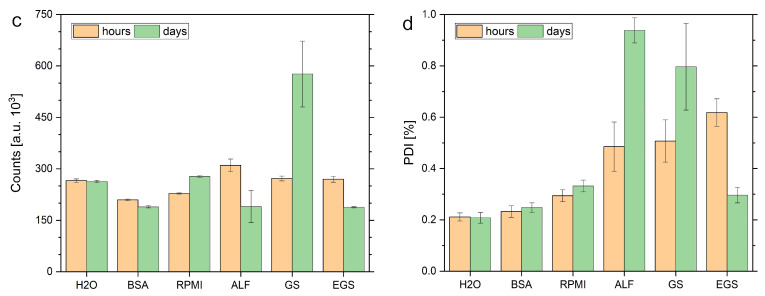
Summary of the mean Z-average size, intensity mean size, polydispersity index (PDI) and counts of the dynamic light scattering measurements of CuO NP dispersions (25 mg L${}^{-1}$, n = 3) in different media (Milli-Q water, BSA solution, cell culture medium (RPMI), artificial lysosomal fluid (ALF), Gamble’s solution (GS) and enhanced Gamble’s solution (EGS)) after about 0, 1, 2, 4, 6, 8 h (left bar, orange) and after 1, 2 and 7 days (right bar, green). (**a**) Provides the calculated hydrodynamic radius of the NP in solution based on the determined z average and (**b**) the mean intensity-based size in the different media. (**c**) Shows the particle counts of the measurements, and (**d**) presents the PDI of the particle size distributions after individual optimization of the measurement conditions of the different media.

An overview of different observed key parameters of DLS measurements of complex media spiked with dispersed CuO NP determined at different time points is presented in Figure 1.

The observed size reflects only the hydrodynamic diameter of the particles inside the samples. Therefore, the observed size corresponds to the overall size of a particle, e.g., their metal/metal oxide and their so-called “corona” formed by matrix components. Furthermore, scattering particles influence the overall results with different contributions based on their size. This is, to a certain extent, reflected in the comparison of the sizes obtained by the z-average compared to the intensity mean-based hydrodynamic radii (see Figure 1). The polydispersity index (PDI) provides a general idea of the complexity of the size distribution of the sample. A PDI value below 0.05 represents a monodisperse sample, while a PDI above 0.7 reflects a broad size distribution of particle characteristics of a polydisperse sample. In combination with the count rate of the respective averaged measurements, these two parameters can be used to evaluate the measurement conditions in general and interpret the obtained results. Changes in the count rate provide a hint about the stability of the particles inside the solution, despite its automatic optimization. An average of the results based on the time points at 0, 1, 2, 4, 6, 8 h (see Figure 1, orange bars) and the results obtained after 1, 2 and 7 days (see Figure 1, green bars) highlights the different behavior of the same amount of CuO NPs inside the different media. The principle behind the size determination leads to challenges of measurements with ALF, GS and EGS matrices. The influence of newly formed or modified particulate content can be determined by significantly higher PDI values and more fluctuating count rates between individual repeats and time points. Another point, which has to be taken into account, is the less stabilizing effect of these matrices, which contributes to the higher standard deviations. These findings indicate changes in the particle content of the GS, ALF and EGS solutions. Due to insufficient particle numbers, the measurements of the blank media could not be used as a reference for background levels of particulate matter formed out of the media itself. In the case of GS, it was reported that the ionic content is not stable over time. This might lead to particle formation and agglomeration of the ionic content. In case of the EGS solution, the decreasing PDI and counts, in combination with the increasing size (intensity-based), can be attributed to the formation of lipid structures, such as liposomes, inside the solution or the formation of bigger NP-lipid clusters. A similar effect could be observed in experiments with gold NPs and liposomes [43]. These agglomerates may be formed over time and enhance the NP agglomeration too. Lastly, the results of the ALF solutions can be explained with a loss of particles derived from the significant decrease in counts between the time points. The pH-dependent dissolution of the CuO NPs in this acidic media points in this direction. This can be observed in the determined sizes based on the intensity mean distributions too. In comparison, the solutions containing water, BSA or RPMI cell culture medium provide similar responses for the size determination approaches with slight changes over time. The PDI shows slight differences between these media, as assumed from their composition. The count rates provide a stable measurement with smaller changes over time. A conclusion might be that the stabilizing effect of the bio-molecules and the buffer solution lead to better working conditions in terms of DLS measurements. With this summary of the results, the advantages of DLS are highlighted, which are to provide a first and fast insight into changes or their absence. A remaining challenge is correlating the observed parameters correctly to the underlying mechanisms. This is especially true for complex samples and matrices, as shown here.

### 3.2. Microwave Digestions of CuO NP Dispersions in Different Media

**Figure 2:**
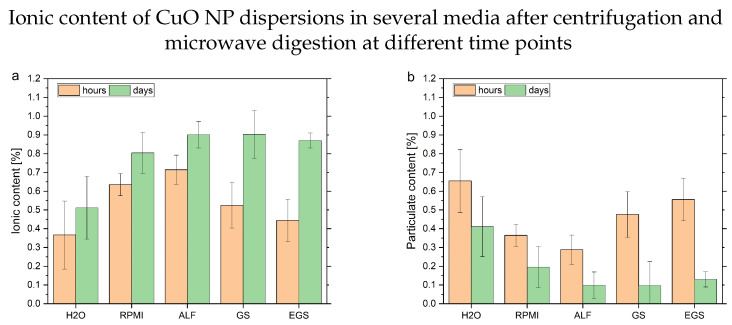
Ionic content of CuO NP dispersions (20 µg L${}^{-1}$, n = 3) in different media after centrifugation and successive microwave digestions normalized to the total ionic content. These values represent the average of the first 8 h and after 1, 2 and 7 days, respectively. (**a**) Depicts the ionic content of the supernatant of the solution and (**b**) the bottom part of the solution after centrifugation, normalized to the total ionic content.

A different approach to dealing with complex media and their potential limiting effects is the separation of NP sample material from dissolved ionic content by centrifugation, the reduction in the matrix by microwave digestion and a successive quantification by ICP-MS of the respective ionic contents. This reduces the burden on the characterization techniques at the cost of a loss of information. To avoid uncertainties due to the additional preparation steps, the individual absolute ionic contents were normalized to the respective total content values. The big advantage of this strategy is its high versatility and robustness. The results presented in Figure 2 show an increase in ionic content in all media, albeit only slightly in water.

**Figure 3:**
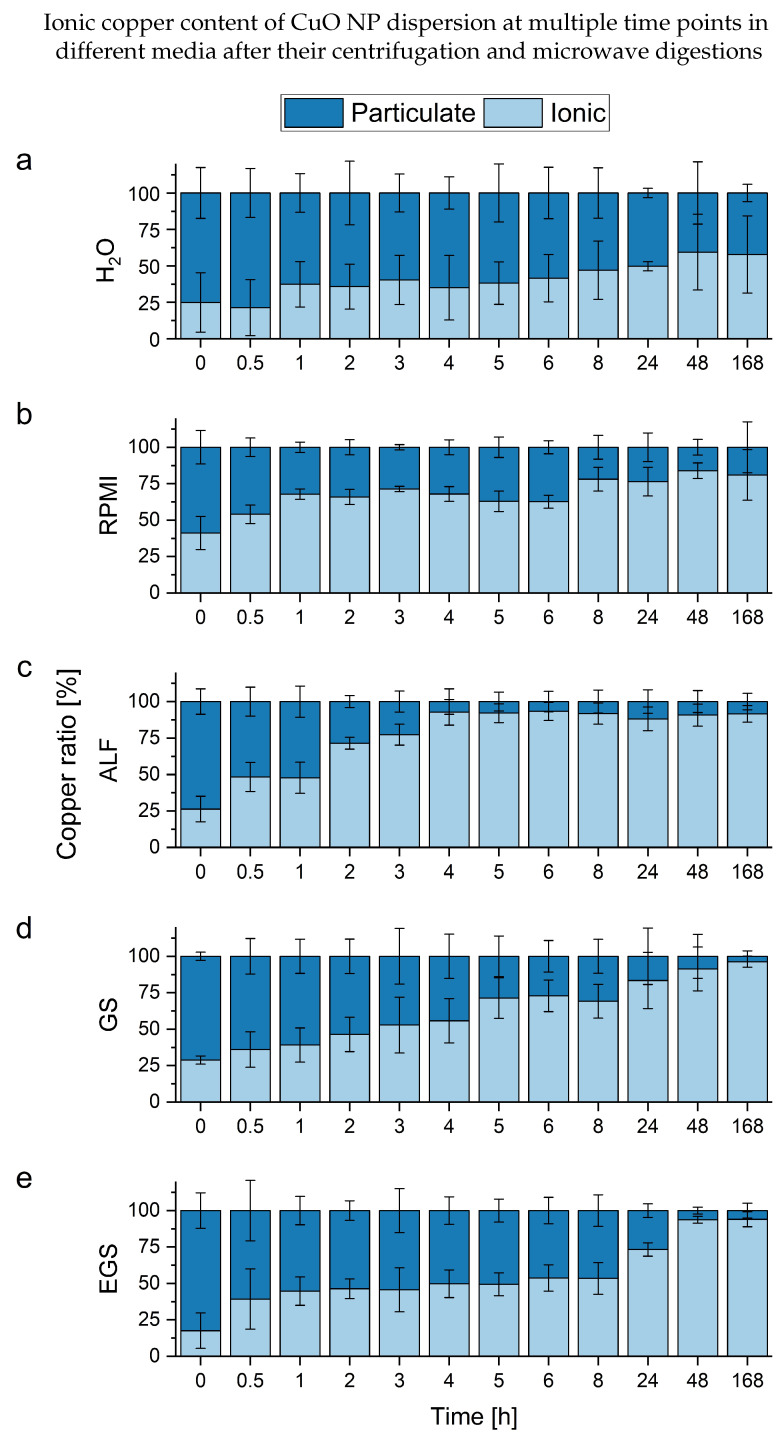
Summary of the measurements of CuO NPs dispersions (20 µg L${}^{-1}$, n = 3) in different media after centrifugation and microwave digestions as a percentage of the total ionic content. The different media are (**a**) deionized water, (**b**) RPMI, (**c**) ALF, (**d**) GS and (**e**) EGS.

Accordingly, the particulate content decreases after 8 h significantly. Due to the sample preparation approach in combination with the ICP-MS characterization, these results reflect different processes compared to the DLS measurement. The internal comparison and interpretation of the time-resolved data allow for a more detailed look into the individual measurements of the different media (see Figure 3). This is possible due to the lower individual uncertainties at the respective time points compared to the DLS measurements.

The trends observed with the DLS measurements are only partly visible due to the different parameters observed. The water solution shows high uncertainties over all time points, which prevents an in-depth interpretation. One point, which needs to be considered, is the additional uncertainty originating from the increased number of working steps. This may lead to a bigger number of experimental errors. Furthermore, the centrifugation-based separation of dissolved and particulate content depends on the density of the NPs and takes time. Both considerations might slightly influence the result toward higher ionic values. However, a slight loss of particulate content can be seen relative to the ionic background values. In contrast, the RPMI-containing samples maintain a stable ratio for several hours after the particles are dispersed homogeneously. A similar pattern can be more clearly observed in the EGS medium. A possible explanation is the stabilizing effect of bigger molecules from the media itself, e.g., proteins and lipids. This is in line with the exclusion of pH effects due to the buffered solutions. In terms of the GS solution, which only contains smaller ionic molecules, a steady increase in ionic content can be observed. Lastly, the fastest dissolution can be seen in ALF. This further verifies the pH-driven dissolution of the CuO NPs in this acidic medium. Due to the reduced matrix influences caused by the microwave digestion, this approach provides a robust way to answer questions about the dissolution of metal NPs in complex media. However, if the characterization of the individual NPs (e.g., size (distribution), shape, number) is required, this method cannot supply sufficient information.

### 3.3. Development of a Direct-Injection Single Particle Inductively-Coupled Plasma Mass Spectrometry System

A method that combines the high sensitivity of the ICP-MS with information about the individual NPs is the spICP-MS. This approach is typically performed in diluted aqueous samples with diluted nitric acid as a carrier to enhance the overall performance. The challenge of this method is based on carry-over effects of the diluted acid due to the autosampler system, as well as the higher necessary sample concentration. These challenges were dealt with with the DI spICP-MS approach and pure water as the carrier solution. First, the feasibility of the DI spICP-MS set-up, despite the different complex matrices and the loss in overall sensitivity due to a non-acidic measurement environment, is evaluated and addressed. This includes a general optimization of the overall set-up and experimental parameters with ionic and particulate standards. Following these experiments, CuO NP dispersions in the complex media were characterized.

**Figure 4:**
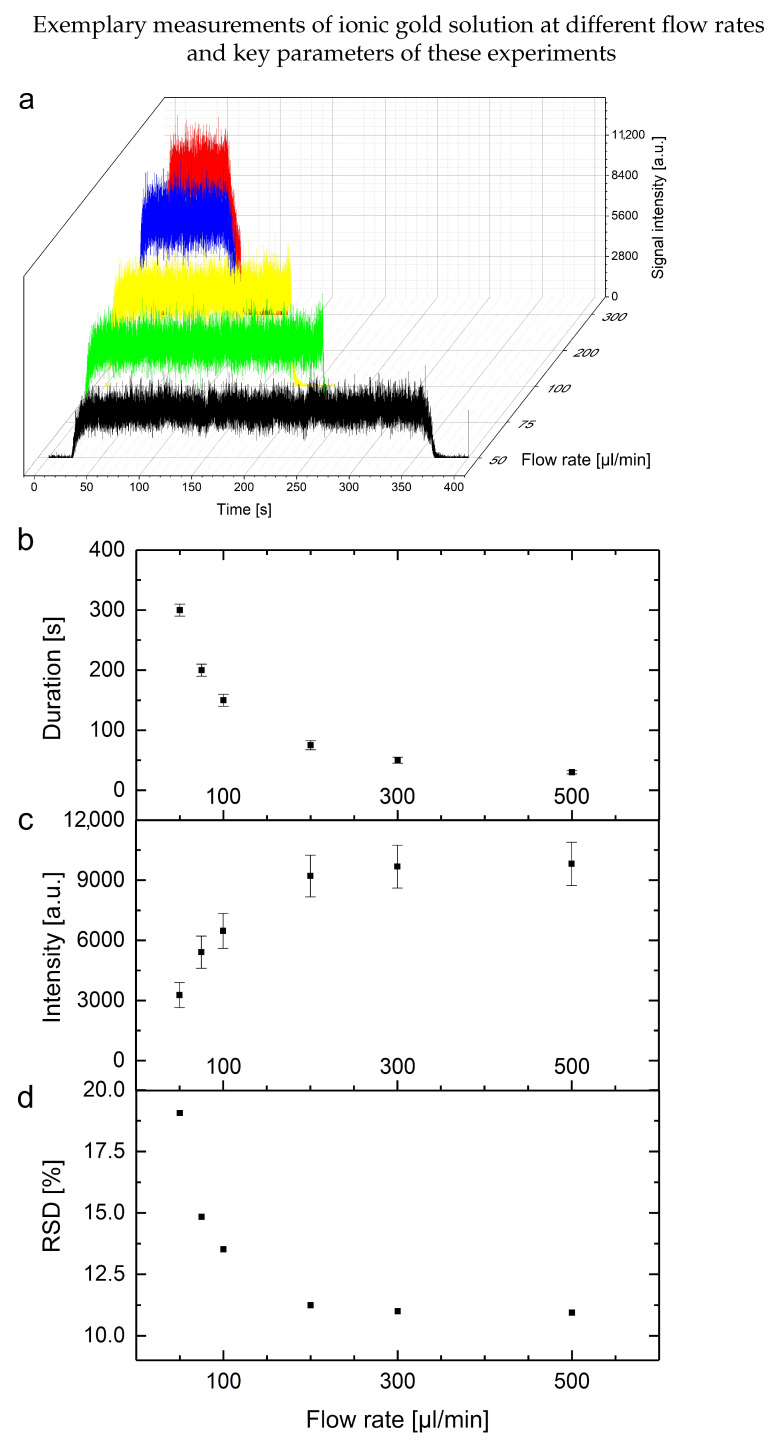
Summary of the observed influences of measurements of ionic gold solutions (5 ppb, n = 3) with different flow rates (50 (black), 75 (green), 100 (yellow), 300 (blue) and 500 µL/min (red)) on the average: (**a**) typical measurements (intensities per time) per colour-coded flow rate, (**b**) duration of the transient signal, (**c**) averaged intensities of the transient signal and (**d**) relative standard deviation of these measurements.

For these measurements, a modified spICP-MS set-up with 2 syringes (5 mL and 500 µL) for exact volume handling, stable performance and fast measurements were used. The challenge of such a direct injection system is the effect of the different undiluted media on the experimental set-up and process parameters. Additionally, matrix effects during the transport, spray, nebulization and ionization processes might further influence the results. Therefore, the goal was a reduction in the necessary sample volume to reduce the burden on the instrument. After the general set-up was configured, further enhancement of signal intensities was achieved due to small amounts of air at the start and end of the sample volume inside the sample loop. With these arrangements, transient signal profiles could be achieved with flow rates between 50 and 500 µL per minute (see Figure 4). The general effects observed with an increasing flow rate are a smaller time frame of the transient signal with an increased signal intensity and a decreased relative standard deviation. In this example, a plateau of those effects is reached above a flow rate of roughly 200 µL/min. However, the viscosity of the different media might inhibit the application of lower flow rates due to clogging of the nebulizer. To have a fast, robust and comparable set of parameters, a flow rate of 500 µL/min was chosen. The downside of this approach is the relative loss of signal intensity compared to the average measurement duration. In theory, this limits the technique to lower NP number concentrations to avoid possible double NP events. A dilution of the sample would solve these issues and reduce the analytical challenges.

A general overview of the results of NP measurements (Au NIST 8012) in the individual media is presented in Table 1. Another significant challenge of spICP-MS is the selection of a limit for the ionic background. This is especially true in the case of a naturally occurring ionic content, dissolving particulate content or small particle sizes, which leads to low spike intensities. To avoid the influence of a subjective determination of this limit, an automated approach was performed. The limit is based on the corrected ionic average and a safety margin of 3 σ or 5 σ RSDs of the individual time frame to calculate the number of NP events. As shown in Table 1, successful measurements and data evaluation could be performed in all media. Due to this mathematical approach, a systematic error can be observed when sizes are compared to the certified size of the particles (approx. 30 nm). This is especially true for the higher safety margin of the 5 σ approach. Therefore, the 3 σ calculations were chosen for the data interpretation of the CuO NP experiments.

Another potential systematic error might be due to ionic background values below the electrical noise. In these cases, an overestimation of the number of events due to these noise values is to be expected. Nonetheless, the observed particle numbers per measurement and general considerations are in line with the recommendation of the ISO/TS 19590:2017 [43]. In conclusion, for a quantitative evaluation, careful calibration and reference measurements are necessary to compensate for these effects. Another option is to further optimize the experimental set-up and parameters for each media, respectively. The 3 σ or 5 σ approach to determine the ionic background levels individually for each time frame and measurement grants an objective way to handle complex cases in an automated, reproducible way. This advantage becomes more pronounced when comparing results over time. Then, the day-to-day performance of the instrument might influence the measurement results significantly, in addition to the present matrix effects.

### 3.4. Nanoparticle Characterization in Complex Media with DI spICP-MS

**Figure 5:**
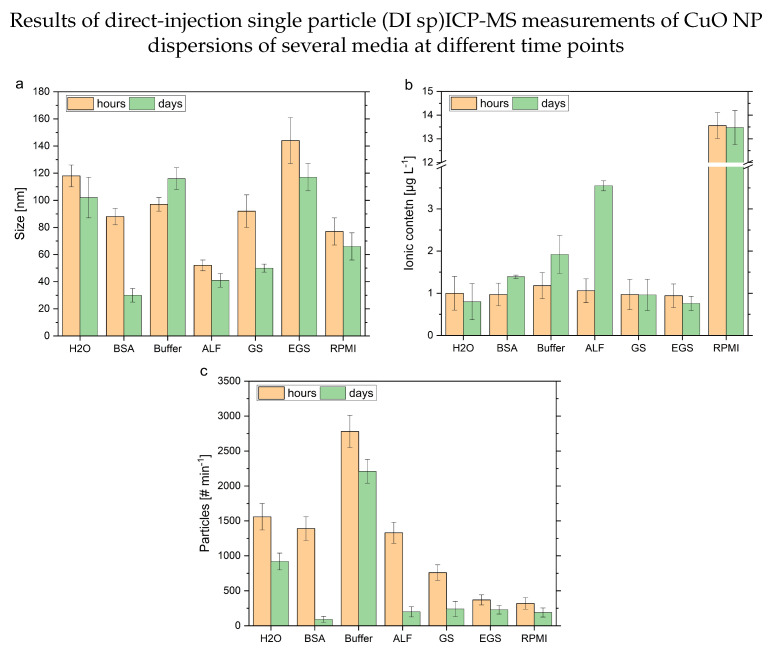
Results of the DI spICP-MS measurements of dispersions of CuO NPs (1.0 µg L${}^{-1}$, n = 3) in different media. The orange bars are the averaged values of the first 3 measurements (a few minutes up to 2 h after dispersion,) and the green bars represent the last 3 measurements (after a week), respectively. The figures are (**a**) the median size of the individual size distributions, (**b**) the determined ionic background levels and (**c**) the observed particle numbers per minute calculated by the 3 σ approach in the different media.

**Figure 6:**
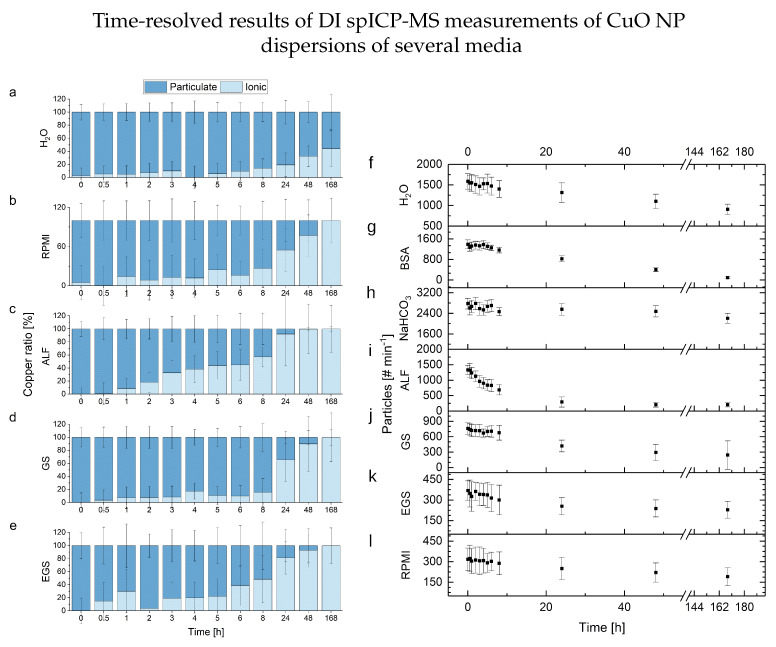
Time-resolved results of the measurements of solutions of CuO NPs (1.0 µg L${}^{-1}$, n = 3) in different media: On the left-hand side (**a**–**e**) is the copper content based on the particulate values determined by the 3 σ approach, and on the right-hand side (**f**–**l**), the observed particle numbers.

To evaluate the presented approach (see Section 3.3) of an objectively evaluated data treatment procedure in combination with a direct-injection measurement method, experiments were performed with the CuO NPs dispersed in a broader variety of media. A summary of these experiments is shown in Table 2 and Figure 5 and Figure 6. In all different media, successful measurements could be carried out. Exemplary time-resolved results of the respective media are provided in the supplementary materials (see Figures S1–S6). Due to the reduced time for each individual measurement, the lower required volumes and the automatized procedure, a better time resolution could be achieved. However, the matrix influences the sensitivity of the measurements, the observed ionic background levels, as well as the calculated transport efficiencies. A comparison with bar plots shows the advantages of this technique (see Figure 5).

The size of the particles based on the time points at the start and after 7 days provides a significant difference in BSA, buffered systems, ALF and GS media. In the case of BSA and RPMI, the number of particles determined after 7 days was below the recommended limit of the ISO/TS 19590:2017; therefore, these values are written in parentheses in Figure 5 [43]. It is important to note that in the ICP-MS-based evaluation, the element-specific information is the basis of the size information. This explains different findings compared to the DLS-determined hydrodynamic sizes in the artificial lung surfactant media. For the pure water, EGS, and cell culture media, no significant change in size could be determined. This is in line with the findings of the DLS measurements. Although, the sizes determined with the DI spICP-MS approach are influenced by matrix effects (see Figure 5, RPMI), which might be attributed to the copper content of the media. The blank media measurements revealed the RPMI as the origin of dissolved copper, leading to a high ionic background. The observed sizes are in most cases significantly higher than the size provided by the manufacturer (<50 nm). Possible reasons are related to the influence of the different media and the size distribution of the CuO NPs. On the one hand, the particle agglomeration itself can be influenced by the constituents of the media. Additionally, matrix effects lead to a reduced sensitivity of the DI spICP-MS. Therefore, smaller-sized NPs might not be detected, and thus, the calculated particle size based on the detected bigger NPs is above the value claimed by the manufacturer. Furthermore, similar to the DLS measurements, this approach provides more information than a calculated spherical size (see Table 2 and Figure 5b,c). The comparison of the ionic content at the two time points shows a stable amount in several dispersions over the week. Nonetheless, the matrix effects strongly influence the sensitivity, which leads to high uncertainties for the DI spICP-MS. In contrast, ALF shows a clear dissolution, with a significantly increased ionic copper content. This behavior is only partly reflected in the observed count rates and PDI of the DLS data (see Figure 1c,d). Finally, the visualization of the particle number concentrations highlights an NP decrease over time. In combination with the determined ionic content of the individual time points, losses can be attributed to dissolution or other unidentified processes In the case of EGS and RPMI, the matrix influences reduce this effect. A more detailed look into the dissolution process was provided with the microwave digestion approach (see Figure 3).

A comparison of these findings with the DI spICP-MS results is provided in Figure 6. Due to the calculation of ionic content based on the determined particle numbers, a significant increase in ionic content attributed to ionic background values can be observed in all media used in the microwave-assisted approach. However, these findings are influenced by matrix effects such as higher background values and lower sensitivity, which cause a higher standard deviation. These effects are reduced due to the digestion and dissolution in the conventional ICP-MS experiments. Nonetheless, the additional information provided by the DI spICP-MS approach, in combination with the reduced efforts, lead to unique applications and insights due to characterized particles inside the media. This can be seen in the right-hand-side graphs of Figure 6, where a visualization of the determined averaged particle numbers at several time points shows different types of kinetics inside the various media. To sum up and generalize these results: the DI spICP-MS approach proves to be a fast and versatile technique to observe metallic NPs directly. This technique, with the systematic data evaluation strategy in combination with the fast measurements, offers a strong tool to determine particle fate. Lastly, detailed optimizations of the individual process parameters can increase the performance further for a certain analytical question (e.g., a specific medium, ionic concentration, particle number or distribution) as shown in Figure 4.

## 4. Conclusions

A key point of this study is the discussion of different techniques and their applicability for different analytical questions with relation to NP characterization. Each technique has its individual advantages and challenges. It is, therefore, important to define which parameters must be investigated at which measurement conditions, including the amount of time and resources available. The analytical question itself includes the differentiation between quantitative and qualitative information and, additionally, the requirements in terms of precision and reproducibility. As a baseline, pure aqueous solution medium was selected. To increase the analytical challenges step-wise, BSA-containing solution and sodium hydrocarbonate buffer were chosen. Due to their application in toxicological experiments, a representative cell culture medium was selected. With regard to experimental dissolution scenarios and their biological relevance, ALF, GS and EGS were used. The observed time frame of 7 days was sufficient to characterize NP behavior. The results of the CuO NP dispersions in pure aqueous media showed a significant loss of particles. The determined sizes and ionic contents over time were in the same range due to the high uncertainty of the measurements. Therefore, the origin of this particle loss cannot be determined. The experiments performed with NP dispersions in BSA-containing media demonstrate a clear loss of NP numbers as well. Additionally, the ionic content increased significantly over time, and the determined NP size decreased. Therefore, the assumption can be made that NP dissolution and agglomeration of bigger particles followed by with their sedimentation took place. Other processes leading to particle loss might have occurred in all media too. The NP dispersions with buffered media showed a less pronounced loss of particles. The observed NP sizes and the ionic content increased slightly. This indicated a slower dissolution due to the avoidance of a pH-driven dissolution mechanism. Furthermore, a significant loss of bigger particles, decreasing the overall particle size, can be excluded. The CuO NP dispersions with the acidic ALF showed a fast loss of particles, a strong increase in ionic content and a slight decrease in particle size. A fitting explanation was the existence of a pH-driven dissolution. However, agglomeration and sedimentation were likely to happen as well. The experiments with the GS media indicated a significant loss of particles, a decrease in size and an increase in ionic content, as shown by the MW ICP-MS data. Similar to the conclusions of the BSA-containing NP dispersions, dissolution and a combination of agglomeration and sedimentation were likely to take place. The experiments of NP dispersion in EGS showed only a slight loss of particles and a decrease in size, while the MW ICP-MS results revealed an increase in ionic content. Here, dissolution took place, but the agglomeration behavior is mitigated compared to the observed behavior of the experiments with the GS media. Lastly, the CuO NP dispersions in RPMI media showed the lowest starting number of particles, and due to the uncertainties, no loss can be determined. However, the digestion experiments revealed a slight dissolution. Due to the same observed sizes, sedimentation processes seem unlikely. An overview of these mentioned aspects and their impact on the applicability of the shown techniques is provided in Table 3. Additional NP traits, which might impact the analysis, such as shape, were neglected here as no further data regarding these topics were collected.

Based on the results presented here, it can be concluded that the DLS measurements act as a fast, low-cost method, albeit unknown matrix influences might be challenging. The microwave digestion proves to be a robust and reliable method at the cost of information loss and time-consuming sample preparation steps. The newly introduced combination of a DI spICP-MS approach with an automatized data evaluation procedure produces fast and reproducible results for a broad range of complex media. Nevertheless, matrices can influence the overall performance and instrumental strain. Therefore, an in-depth optimization must be performed for a quantitative characterization. Further technical improvements or adapted configurations may lead to a higher precision or multi-elemental analysis. This approach is especially promising in differentiating between ionic-based observed toxicity effects of metallic NPs and particulate-driven processes. This is exemplarily shown in the acidic scenario of the ALF compared to the other matrices. This new information could expand the knowledge about ongoing NP-related processes in different environments and allows the development and utilization of less toxic NP.

## Figures and Tables

**Table 1:** Characterization of the calibrations with matrix-matched Au NP dispersions (NIST 8012, 50 ng L${}^{-1}$, n = 3).

Media	H${}_{2}$O	BSA	NaHCO${}_{3}$	ALF	GS	EGS	RPMI
Total content [ng L${}^{-1}$]	52.4 ± 3.1	48.1 ± 5.6	45.3 ± 6.1	60.4 ± 9.8	57.7 ± 13.5	52.4 ± 6.0	44.5 ± 4.2
RSD [%]	6	12	14	16	23	11	10
				3 σ approach			
Particles [min${}^{-1}$]	3580	2230	3200	390	550	1410	520
RSD [%]	6	20	22	11	20	6	9
Transport efficiency	0.0291	0.0187	0.0264	0.0031	0.0068	0.0123	0.0042
Particle size [nm]	27 ± 2	28 ± 1	30 ± 1	24 ± 1	26 ± 1	26 ± 1	25 ± 1
				5 σ approach			
Particles [min${}^{-1}$]	214	943	152	31	63	126	33
RSD [%]	33	16	20	28	21	24	22
Transport efficiency	0.0018	0.0083	0.0013	0.0003	0.0006	0.0010	0.0003
Particle size [nm]	15 ± 1	27 ± 1	15 ± 1	9 ± 2	8 ± 3	14 ± 1	10 ± 2

**Table 2:** Characterization of CuO NP dispersions (1 µg L${}^{-1}$, n = 9) in different media after 1 h and 7 days calculated with the 3 σ approach.

Media	H${}_{2}$O	BSA	NaHCO${}_{3}$	ALF	GS	EGS	RPMI
				Start			
Total content [µg L${}^{-1}$]	1.00 ± 0.40	0.97 ± 0.27	1.18 ± 0.31	1.06 ± 0.28	0.97 ± 0.36	0.94 ± 0.28	13.56 ± 0.55
Particles [min${}^{-1}$]	1560 ± 190	1390 ± 170	2780 ± 230	1330 ± 150	760 ± 112	370 ± 72	320 ± 83
Particle size [nm]	118 ± 8	88 ± 6	97 ± 5	52 ± 4	92 ± 12	144 ± 17	77 ± 10
				End			
Total content [µg L${}^{-1}$]	0.80 ± 0.43	1.39 ± 0.04	1.92 ± 0.45	3.55 ± 0.12	0.96 ± 0.37	0.76 ± 0.17	13.47 ± 0.72
Particles [min${}^{-1}$]	910 ± 120	(*92 ± 44*)	2210 ± 170	200 ± 73	240 ± 110	230 ± 62	(*190 ± 65*)
Particle size [nm]	102 ± 15	(*30 ± 5*)	116 ± 8	41 ± 5	50 ± 3	117 ± 10	(*66 ± 10*)

**Table 3:** Overview of different analytical aspects of the DLS, microwave-assisted (MW) ICP-MS, spICP-MS and DI spICP-MS experiments.

	DLS	MW ICP-MS	(DI) spICP-MS
Sample preparation	minutes	hours	minutes
Sample amount	mL	mL	mL–µL
Sensitivity (NP content)	low	high	high
Sensitivity (NP size)	high	low	medium
Robustness	low	high	medium
Data Interpretation	medium	low	medium

## Data Availability

The data presented in this study are available upon justified request.

## Supplementary Materials

The following are available online at https://www.mdpi.com/article/10.3390/nano13050922/s1, Figure S1: Time-resolved DI spICP-MS analysis of CuO NP dispersions (1 µg/L) in aqueous media.; Figure S2: Time-resolved DI spICP-MS analysis of CuO NP dispersions (1 µg/L) in BSA; Figure S3: Time-resolved DI spICP-MS analysis of CuO NP dispersions (1 µg/L) in NaHCO${}_{3}$; Figure S4: Time-resolved DI spICP-MS analysis of CuO NP dispersions (1 µg/L) in ALF; Figure S5: Time-resolved DI spICP-MS analysis of CuO NP dispersions (1 µg/L) in GS; Figure S6: Time-resolved DI spICP-MS analysis of CuO NP dispersions (1 µg/L) in EGS; Figure S7: Time-resolved DI spICP-MS analysis of CuO NP dispersions (1 µg/L) in RPMI.

## Abbreviations

The following abbreviations are used in this manuscript:

NM	nanomaterial
NP	nanoparticle
DLS	dynamic light scattering
sp	single particle
ICP-MS	inductively-coupled mass spectrometry
BSA	Bovine Serum Albumin
RPMI	Roswell Park Memorial Institute
GS	Gamble’s solution
EGS	enhanced Gamble’s solution
ALF	artificial lysosomal fluid
DI	direct-injection
RSD	relative standard deviation
PDI	polydispersity index

## References

[1] Lu A.H., Salabas E.L., Schuth F. (2007). Magnetic nanoparticles: Synthesis, protection, functionalization, and application. Angew. Chem. Int. Ed..

[2] Medintz I.L., Uyeda H.T., Goldman E.R., Mattoussi H. (2005). Quantum dot bioconjugates for imaging, labelling and sensing. Nat. Mater..

[3] Kelly K.L., Coronado E., Zhao L.L., Schatz G.C. (2003). The optical properties of metal nanoparticles: The influence of size, shape, and dielectric environment. J. Phys. Chem. B.

[4] Daniel M.C., Astruc D. (2004). Gold nanoparticles: Assembly, supramolecular chemistry, quantum-size-related properties, and applications toward biology, catalysis, and nanotechnology. Chem. Rev..

[5] Prabhu S., Poulose E.K. (2012). Silver nanoparticles: Mechanism of antimicrobial action, synthesis, medical applications, and toxicity effects. Int. Nano Lett..

[6] Zeng C., Nguyen C., Boitano S., Field J.A., Shadman F., Sierra-Alvarez R. (2021). Toxicity of abrasive nanoparticles (SiO_2_, CeO_2_, and Al_2_O_3_) on Aliivibrio fischeri and human bronchial epithelial cells (16HBE14o-). J. Nanoparticle Res..

[7] De Almeida G.H.G., Siqueira-Soares R.D., Mota T.R., de Oliveira D.M., Abrahao J., Foletto-Felipe M.D., dos Santos W.D., Ferrarese O., Marchiosi R. (2021). Aluminum oxide nanoparticles affect the cell wall structure and lignin composition slightly altering the soybean growth. Plant Physiol. Biochem..

[8] Garcia-Salvador A., Katsumiti A., Rojas E., Aristimuno C., Betanzos M., Martinez-Moro M., Moya S.E., Goni-de-Cerio F. (2021). A Complete In Vitro Toxicological Assessment of the Biological Effects of Cerium Oxide Nanoparticles: From Acute Toxicity to Multi-Dose Subchronic Cytotoxicity Study. Nanomaterials.

[9] Wang Z., Katsumiti A., von dem Bussche A., Kabadi P.K., Kane A.B., Hurt R.H. (2013). Biological and Environmental Transformations of Copper-Based Nanomaterials. ACS Nano.

[10] Duncan T.V. (2011). Applications of nanotechnology in food packaging and food safety: Barrier materials, antimicrobials and sensors. J. Colloid Interface Sci..

[11] Ye Z.P., Li S.Y., Zhao S.Y., Deng L.D., Zhang J.H., Dong A.J. (2021). Textile coatings configured by double-nanoparticles to optimally couple superhydrophobic and antibacterial properties. Chem. Eng. J..

[12] Raj S.N., Anooj E.S., Rajendran K., Vallinayagam S. (2021). A comprehensive review on regulatory invention of nano pesticides in Agricultural nano formulation and food system. J. Mol. Struct..

[13] Aziz Z.A.A., Mohd-Nasir H., Ahmad A., Mohd Setapar S.H., Peng W.L., Cgui S.C., Khatoon A., Umar K., Yaqoob A.A., Mohamad Ibrahim M.N. (2019). Role of Nanotechnology for Design and Development of Cosmeceutical: Application in Makeup and Skin Care. Front. Chem..

[14] Chen S., Zhang Q., Hou Y., Zhang J., Liang X.-J. (2013). Nanomaterials in medicine and pharmaceuticals: Nanoscale materials developed with less toxicity and more efficacy. Eur. J. Nanomed..

[15] Valsami-Jones E., Lynch I. (2015). How safe are nanomaterials?. Science.

[16] He X., Deng H., Hwang H.-M. (2019). The current application of nanotechnology in food and agriculture. J. Food Drug Anal..

[17] Schulte P.A., Leso V., Niang M., Iavicoli I. (2019). Current state of knowledge on the health effects of engineered nanomaterials in workers: A systematic review of human studies and epidemiological investigations. Scand. J. Work Environ. Health.

[18] Leitner J., Sedmidubský D., Jankovský O. (2019). Size and Shape-Dependent Solubility of CuO Nanostructures. Materials.

[19] Misra S., Nuseibeh S., Dybowska A., Tetley T., Berhanu D., Valsami-Jones E. (2013). Comparative study using spheres, rods and spindle-shaped nanoplatelets on dispersion stability, dissolution and toxicity of CuO nanomaterials. Nanotoxicology.

[20] Amigoni L., Salvioni L., Sciandrone B., Giustra M., Pacini C., Tortora P., Prosperi D., Colombo M., Regonesi M.E. (2021). Impact of Tuning the Surface Charge Distribution on Colloidal Iron Oxide Nanoparticle Toxicity Investigated in *Caenorhabditis elegans*. Nanotoxicology.

[21] Fazel A.M., Chupani L., Guo Z., Zhang P., Darbha G.K., Vijver M.G., Valsami-Jones E., Peijnenburg W.J.G.M. (2021). The stochastic association of nanoparticles with algae at the cellular level: Effects of NOM, particle size and particle shape. Ecotoxicol. Environ. Saf..

[22] Briffa S.M., Lynch I., Hapiuk D., Valsami-Jones E. (2019). Physical and chemical transformations of zirconium doped ceria nanoparticles in the presence of phosphate: Increasing realism in environmental fate and behaviour experiments. Environ. Pollut..

[23] Conway J.R., Adeleye A.S., Gardea-Torresdey J., Keller A.A. (2015). Aggregation, Dissolution, and Transformation of Copper Nanoparticles in Natural Waters. Environ. Sci. Technol..

[24] Zhao Z., Li G., Liu Q.S., Liu W., Qu G., Hu L., Long Y., Cai Z., Zhao X., Jiang G. (2021). Identification and interaction mechanism of protein corona on silver nanoparticles with different sizes and the cellular responses. J. Hazard. Mater..

[25] Sukhanova A., Bozrova S., Sokolov P., Berestovoy M., Karaulov A., Nabiev I. (2018). Dependence of Nanoparticle Toxicity on Their Physical and Chemical Properties. Nanoscale Res. Lett..

[26] Scola S., Blasco J., Campana O. (2021). “Nanosize effect” in the metal-handling strategy of the bivalve Scrobicularia plana exposed to CuO nanoparticles and copper ions in whole-sediment toxicity tests. Sci. Total Environ..

[27] Ohle J., Witt B., Hartwig A. (2014). Cytotoxicity and genotoxicity of nano - and microparticulate copper oxide: Role of solubility and intracellular bioavailability. Part. Fibre Toxicol..

[28] Vimbela G.V., Ngo S.M., Fraze C., Yang L., Stout D.A. (2017). Antibacterial properties and toxicity from metallic nanomaterials. Int. J. Nanomed..

[29] Misra S.K., Dybowska A., Berhanu D., Luoma S.N., Valsami-Jones E. (2012). The complexity of nanoparticle dissolution and its importance in nanotoxicological studies. Sci. Total Environ..

[30] Stetefeld J., McKenna S.A., Patel T.R. (2016). Dynamic light scattering: A practical guide and applications in biomedical sciences. Biophys. Rev..

[31] Marucco A., Aldieri E., Leinardi R., Bergamaschi E., Riganti C., Fenoglio I. (2019). Applicability and Limitations in the Characterization of Poly-Dispersed Engineered Nanomaterials in Cell Media by Dynamic Light Scattering (DLS). Materials.

[32] Degueldre C., Favarger P.Y., Wold S. (2006). Gold colloid analysis by inductively coupled plasma-mass spectrometry in a single particle mode. Anal. Chim. Acta.

[33] Lee S., Bi X., Reed R.B., Ranville J.F., Herckes P., Westerhoff P. (2014). Nanoparticle Size Detection Limits by Single Particle ICP-MS for 40 Elements. Environ. Sci. Technol..

[34] Peters R., Herrera-Rivera Z., Undas A., van der Lee M., Marvin H., Bouwmeester H., Weigel S. (2015). Single particle ICP-MS combined with a data evaluation tool as a routine technique for the analysis of nanoparticles in complex matrices. J. Anal. At. Spectrom..

[35] Krause B.C., Kriegel F.L., Rosenkranz D., Dreiack N., Tentschert J., Jungnickel H., Jalili P., Fessard V., Laux P., Luch A. (2020). Aluminum and aluminum oxide nanomaterials uptake after oral exposure—A comparative study. Sci. Rep..

[36] Wei W.-J., Li L., Gao Y.-P., Wang Q., Zhou Y.-Y., Liu X., Yang Y. (2021). Enzyme digestion combined with SP-ICP-MS analysis to characterize the bioaccumulation of gold nanoparticles by mustard and lettuce plants. Sci. Total Environ..

[37] Tharaud M., Louvat P., Benedetti M.F. (2021). Detection of nanoparticles by single-particle ICP-MS with complete transport efficiency through direct nebulization at few-microlitres-per-minute uptake rates. Anal. Bioanal. Chem..

[38] OECD (2017). Test No. 318: Dispersion Stability of Nanomaterials in Simulated Environmental Media.

[39] Ji Z., Jin X., George S., Xia T., Meng H., Wang X., Suarez E., Zhang H., Hoek E.M.V., Godwin H. (2010). Dispersion and Stability Optimization of TiO2 Nanoparticles in Cell Culture Media. Environ. Sci. Technol..

[40] Moore T.L., Rodriguez-Lorenzo L., Hirsch V., Balog S., Urban D., Jud C., Rothen-Rutishauser B., Lattuada M., Petri-Fink A. (2015). Nanoparticle colloidal stability in cell culture media and impact on cellular interactions. Chem. Soc. Rev..

[41] Marques M.R.C., Loebenberg R., Almukainzi M. (2011). Simulated Biological Fluids with Possible Application in Dissolution Testing. Dissolution Technol..

[42] He H., Zou Z., Wang B., Xu G., Chen C., Qin X., Yu C., Zhang J. (2020). Copper Oxide Nanoparticles Induce Oxidative DNA Damage and Cell Death via Copper Ion-Mediated P38 MAPK Activation in Vascular Endothelial Cells. Int. J. Nanomed..

[43] Hachenberger Y.U., Rosenkranz D., Kriegel F.L., Krause B., Matschaß R., Reichardt P., Tentschert J., Laux P., Jakubowski N., Panne U. (2020). Tackling Complex Analytical Tasks: An ISO/TS-Based Validation Approach for Hydrodynamic Chromatography Single Particle Inductively Coupled Plasma Mass Spectrometry. Materials.

[44] Kriegel F.L., Reichardt P., Krause B.-C., Sing A.V., Tentschert J., Laux P., Jungnuckel H., Luch A. (2019). The Vitamin A and D Exposure of Cells Affects the Intracellular Uptake of Aluminum Nanomaterials and its Agglomeration Behavior: A Chemo-Analytic Investigation. Int. J. Mol. Sci..

[45] Laborda F., Jimenez-Lamana J., Bolea E. (2013). Critical considerations for the determination of nanoparticle number concentrations, size and number size distributions by single particle ICP-MS. J. Anal. At. Spectrom..

[46] Tuoriniemi G., Cornelis G., Hassellöv M. (2012). Size Discrimination and Detection Capabilities of Single-Particle ICP MS for Environmental Analysis of Silver Nanoparticles. Anal. Chem..

