# Supplementary material for: Nanomaterial Characterization in Complex Media—Guidance and Application
Source: Nanomaterials (Basel). 2023 Mar 2;13(5):922. doi: 10.3390/nano13050922 (PMC10005142; doi:10.3390/nano13050922)
Supplement: Supplementary file 1 [file nanomaterials-13-00922-s001.zip › nanomaterials-2213897-supplementary.pdf]

nanomaterials

Supplementary Materials

Nanomaterial Characterization in Complex Media—Guidance and Application

Yves Uwe Hachenberger [1,†] , Daniel Rosenkranz [2,†] , Charlotte Kromer [1] , Benjamin Christoph Krause [1,†] , Nadine Dreiack [1] , Fabian Lukas Kriegel [1,†] , Ekaterina Koz'menko [1,†], Harald Jungnickel [1] , Jutta Tentschert [1] , Frank Stefan Bierkandt [1,*] , Peter Laux [1,*] , Ulrich Panne [3] and Andreas Luch [1]

[1] Department of Chemical & Product Safety, German Federal Institute for Risk Assessment (BfR), Max-Dohrn-Strasse 8-10, 10589 Berlin, Germany
[2] Institute for Clinical Chemistry and Laboratory Medicin, Klinikum Oldenburg AöR, Rahel-Straus-Straße 10, 26133 Oldenburg, Germany
[3] Federal Institute for Materials Research and Testing (BAM), Richard-Willstätter-Strasse 11, 12489 Berlin, Germany
* Correspondence: frank.bierkandt@bfr.bund.de (F.S.B.); peter.laux@bfr.bund.de (P.L.)
† Former Employee of BfR or/and BAM.

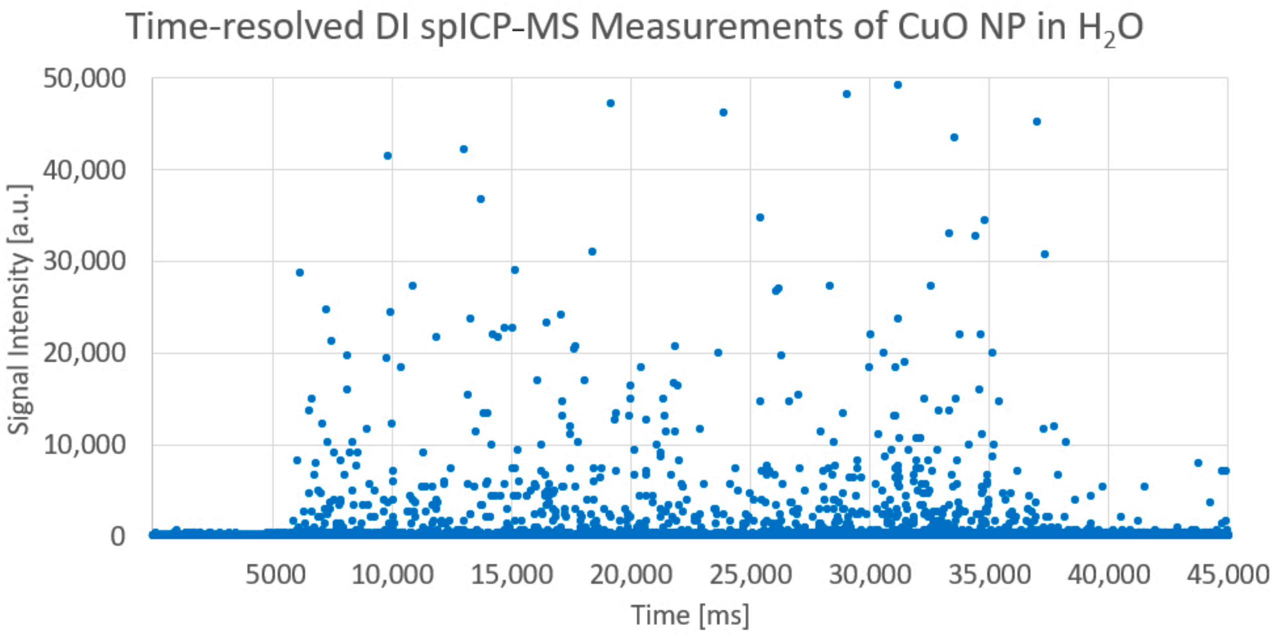

Figure S1. Time-resolved DI spICP-MS analysis of CuO NP dispersions (1 µg/L) in aqueous media.

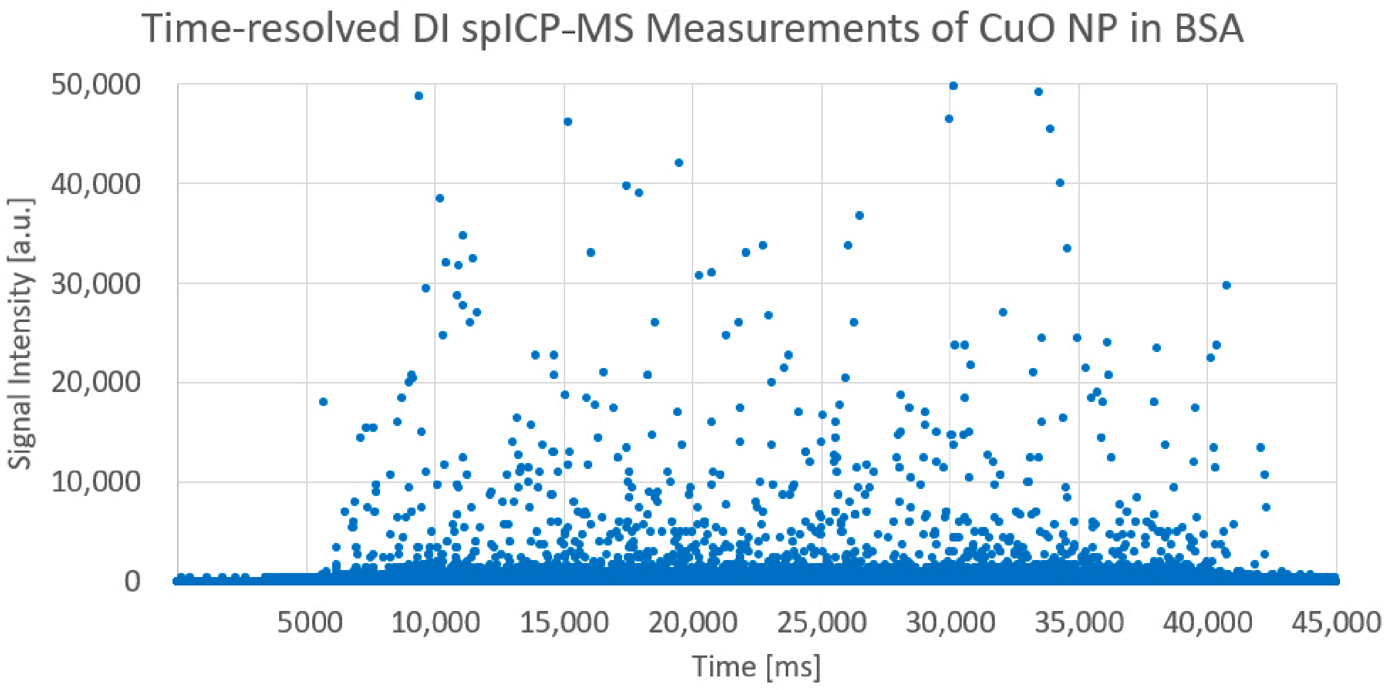

Figure S2. Time-resolved DI spICP-MS analysis of CuO NP dispersions (1 μg/L) in BSA.

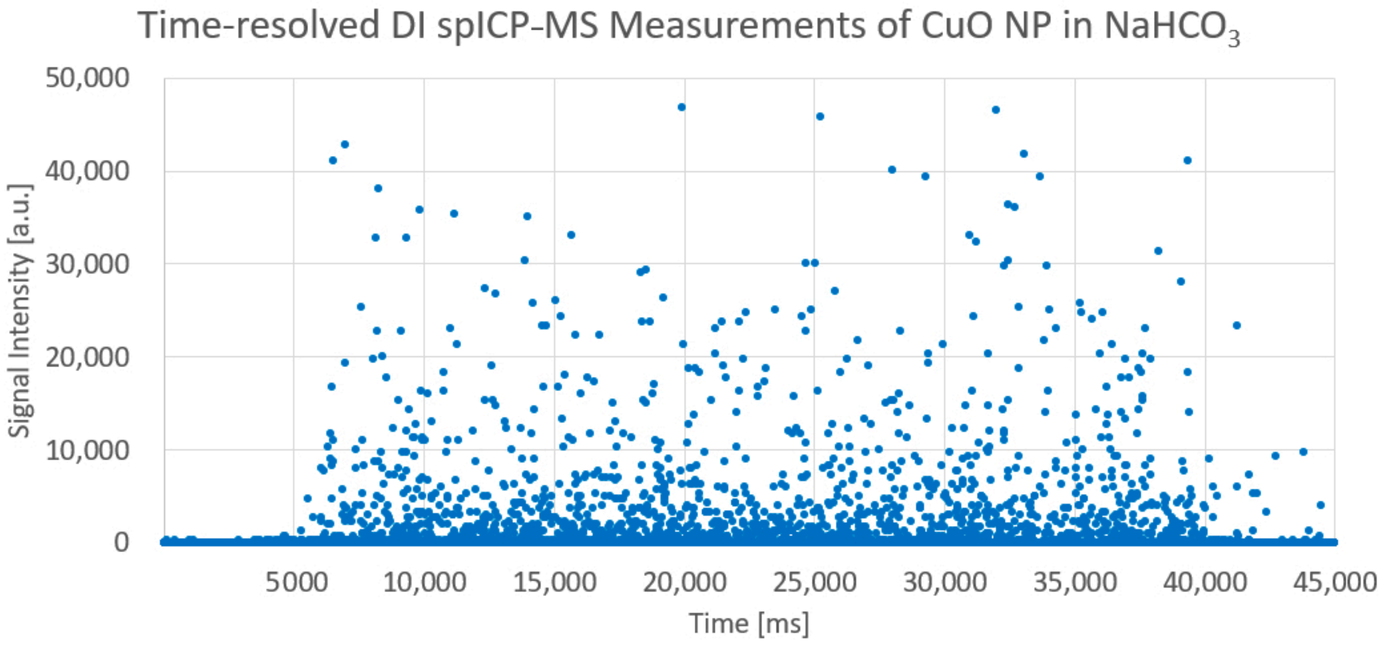

Figure S3. Time-resolved DI spICP-MS analysis of CuO NP dispersions (1 μg/L) in $NaHCO_3$.

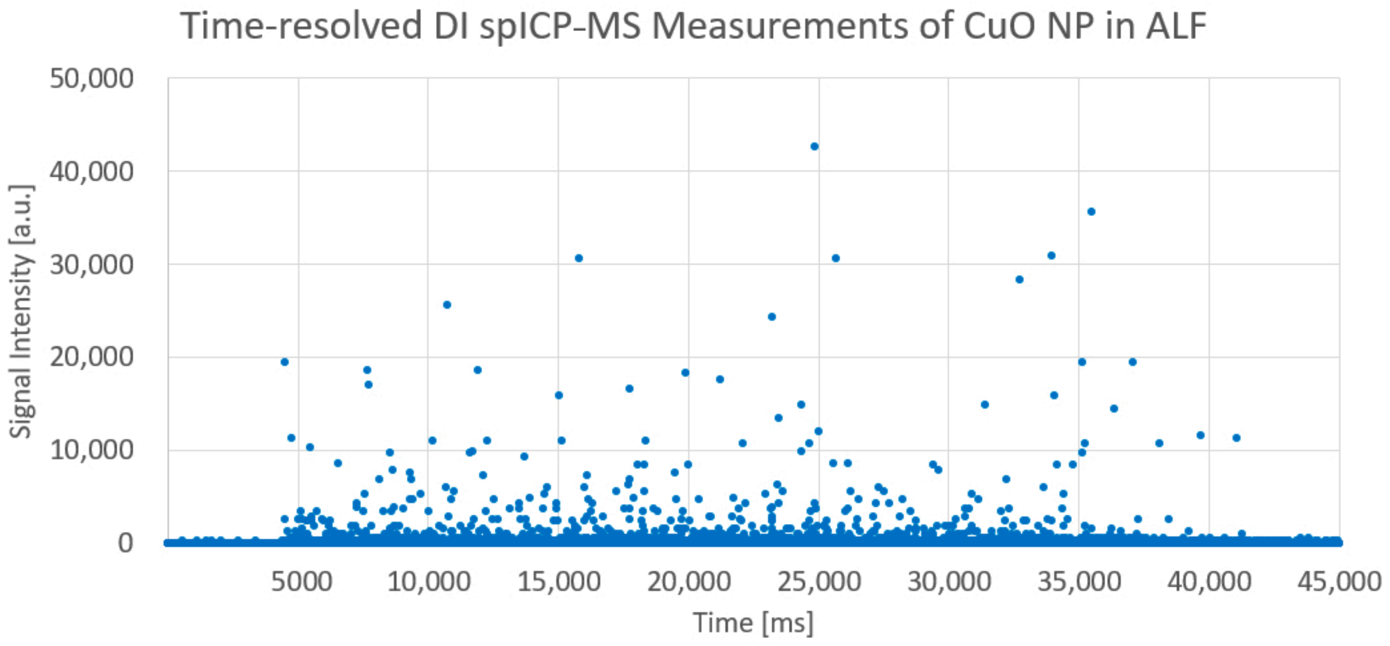

Figure S4. Time-resolved DI spICP-MS analysis of CuO NP dispersions (1 µg/L) in ALF.

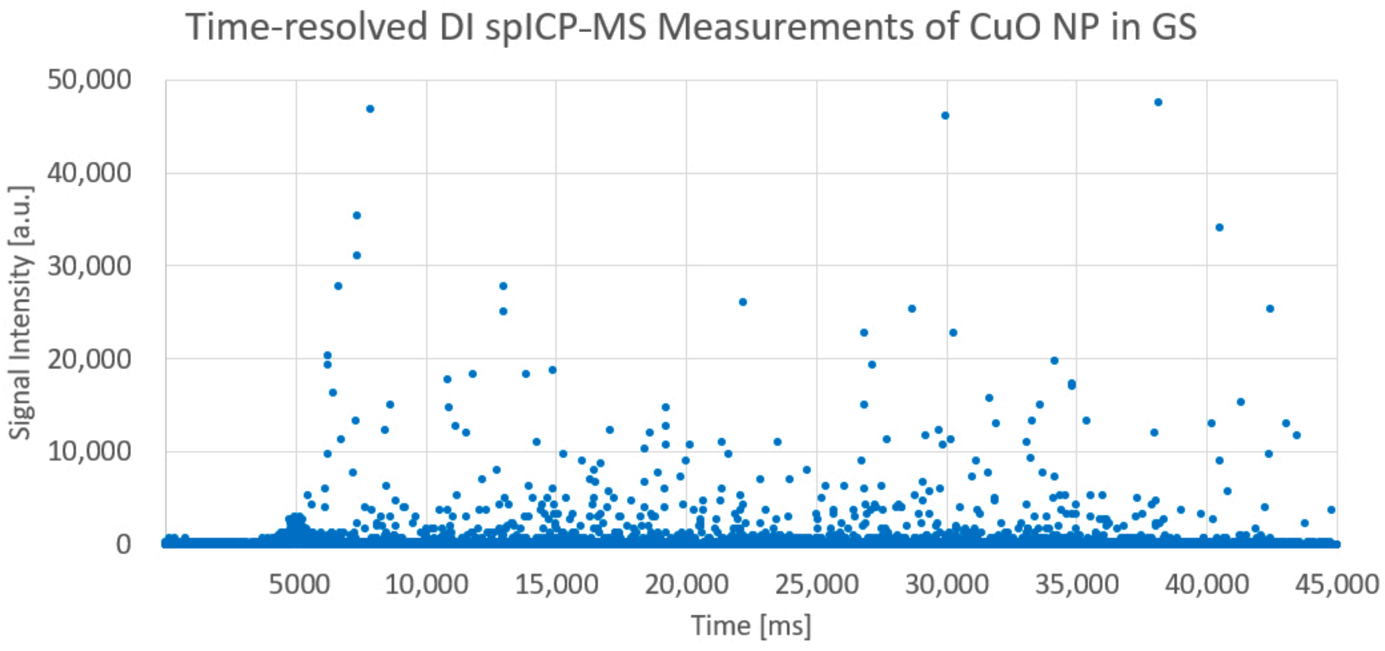

Figure S5. Time-resolved DI spICP-MS analysis of CuO NP dispersions (1 µg/L) in GS.

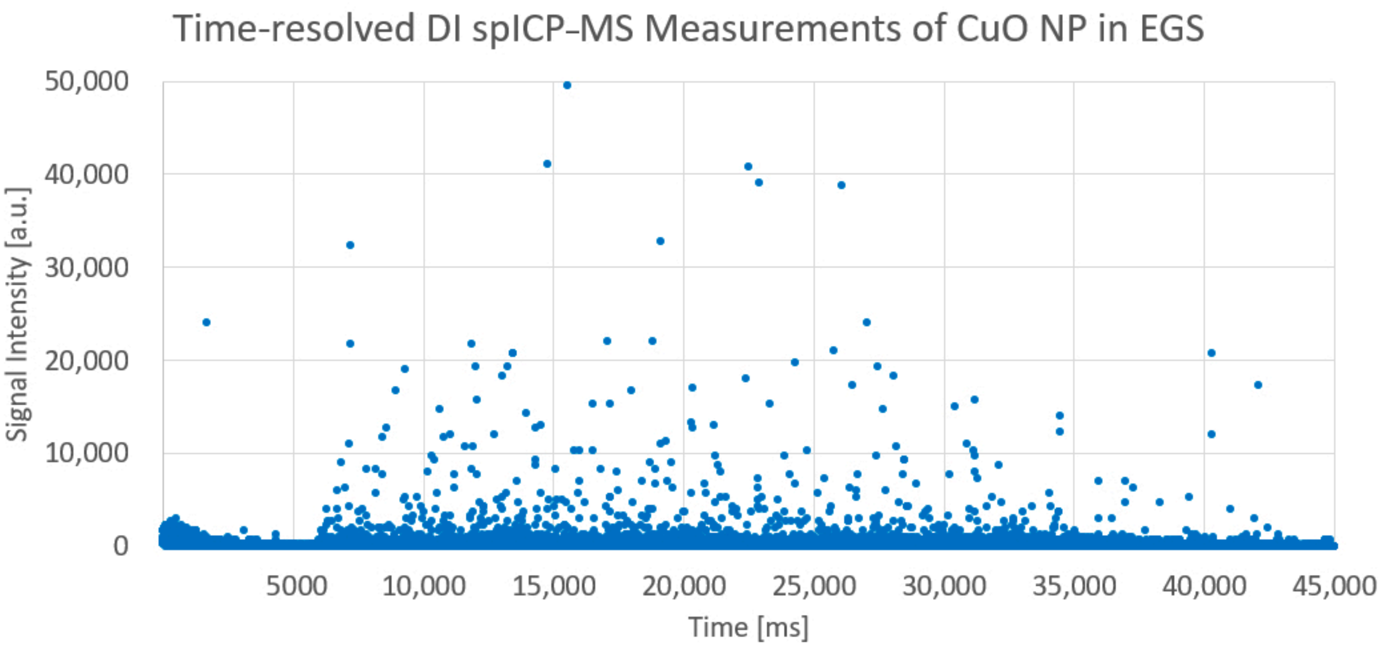

Figure S6. Time-resolved DI spICP-MS analysis of CuO NP dispersions (1 µg/L) in EGS.

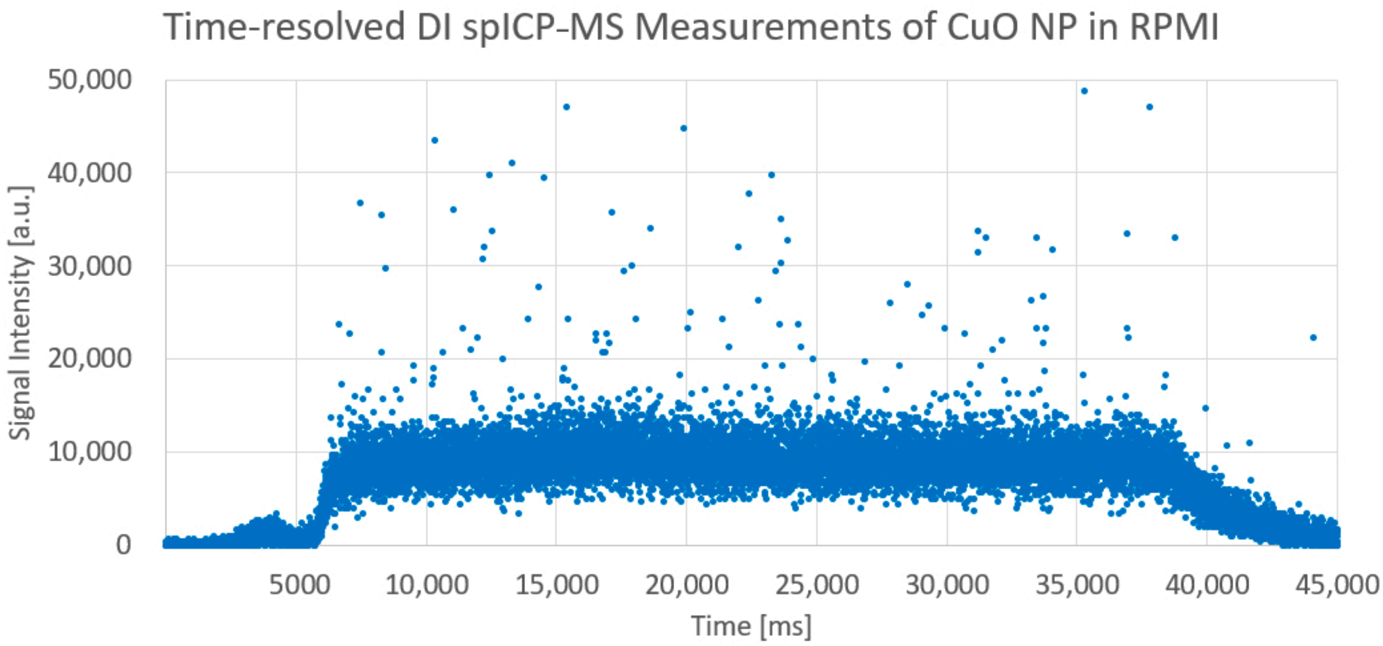

Figure S7. Time-resolved DI spICP-MS analysis of CuO NP dispersions (1 µg/L) in RPMI.
